# The Impact of Combined Warm and Cold Ischemia Time on Post-transplant Outcomes

**DOI:** 10.1177/20543581231178960

**Published:** 2023-06-11

**Authors:** Martha E. Foley, Amanda J. Vinson, Thomas A. A. Skinner, Bryce A. Kiberd, Karthik K. Tennankore

**Affiliations:** 1 Faculty of Medicine, Dalhousie University, Halifax, NS, Canada; 2 Nova Scotia Health, Halifax, Canada; 3 Division of Nephrology, Department of Medicine, Dalhousie University, Halifax, Canada; 4 Department of Urology, Dalhousie University, Halifax, NS, Canada

**Keywords:** kidney transplant, cold ischemia, warm ischemia, graft failure, delayed graft function

## Abstract

**Background:**

Prolonged warm ischemia time (WIT) and cold ischemia time (CIT) are independently associated with post-transplant graft failure; their combined impact has not been previously studied. We explored the effect of combined WIT/CIT on all-cause graft failure following kidney transplantation.

**Methods:**

The Scientific Registry of Transplant Recipients was used to identify kidney transplant recipients from January 2000 to March 2015 (after which WIT was no longer separately reported), and patients were followed until September 2017. A combined WIT/CIT variable (excluding extreme values) was separately derived for live and deceased donor recipients using cubic splines; for live donor recipients, the reference group was WIT 10 to <23 minutes and CIT >0 to <0.42 hours, and for deceased donor recipients the WIT was 10 to <25 minutes and CIT 1 to <7.75 hours. The adjusted association between combined WIT/CIT and all-cause graft failure (including death) was analyzed using Cox regression. Secondary outcomes included delayed graft function (DGF).

**Results:**

A total of 137 125 recipients were included. For live donor recipients, patients with prolonged WIT/CIT (60 to ≤120 minutes/3.04 to ≤24 hours) had the highest adjusted hazard ratio (HR) for graft failure (HR = 1.61, 95% confidence interval [CI] = 1.14-2.29 relative to the reference group). For deceased donor recipients, a WIT/CIT of 63 to ≤120 minutes/28 to ≤48 hours was associated with an adjusted HR of 1.35 (95% CI = 1.16-1.58). Prolonged WIT/CIT was also associated with DGF for both groups although the impact was more driven by CIT.

**Conclusions:**

Combined WIT/CIT is associated with graft loss following transplantation. Acknowledging that these are separate variables with different determinants, we emphasize the importance of capturing WIT and CIT independently. Furthermore, efforts to reduce WIT and CIT should be prioritized.

## Abrégé

**Contexte:**

La période prolongée d’ischémie à chaud (WIT—*warm ischemia time*) et la période prolongée d’ischémie à froid (CIT—*cold ischemia time*) ont été associées de façon indépendante à une défaillance du greffon post-transplantation, mais leur effet combiné n’a jamais été étudié. Nous avons examiné l’effet combiné WIT/CIT sur la défaillance du greffon toutes causes confondues après une transplantation rénale.

**Méthodologie:**

Le Scientific Registry of Transplant Recipients a été utilisé pour identifier les receveurs d’une greffe de rein entre janvier 2000 et mars 2015 (date après laquelle la WIT n’a plus été rapportée séparément). Les patients ont été suivis jusqu’en septembre 2017. Une variable combinée WIT/CIT (excluant les valeurs extrêmes) a été dérivée de façon isolée pour les donneurs vivants et les donneurs décédés à l’aide d’une fonction spline cubique. La WIT du groupe référence pour les donneurs vivants se situait entre 10 et <23 minutes, et la CIT entre 0 et <0,42 heure; pour les donneurs décédés, la WIT se situait entre 10 et <25 minutes, et la CIT entre 1 et <7,75 heures. L’association corrigée entre une combinaison WIT/CIT et la défaillance du greffon toutes causes confondues (y compris le décès) a été analysée à l’aide de la régression de Cox. Les résultats secondaires incluaient une reprise retardée de la fonction du greffon (RRFG).

**Résultats:**

Un total de 137 125 receveurs d’un rein a été inclus. Dans le groupe des receveurs d’un organe provenant d’un donneur vivant, les patients avec une WIT/CIT prolongée (60 à ≤120 minutes/3,04 à ≤24 heures) présentaient un risque relatif corrigé plus élevé de défaillance du greffon (RRc: 1,61; IC 95 %: 1,14-2,29) par rapport au groupe de référence. Dans le groupe des receveurs d’un organe provenant d’un donneur décédé, une combinaison WIT/CIT de 63 à ≤120 minutes/28 à ≤48 heures a été associée à un RRc de 1,35 (IC 95 %: 1,16-1,58). La WIT/CIT prolongée a également été associée à une RRFG pour les deux groupes, bien que cet effet ait été davantage influencé par la CIT.

**Conclusion:**

La combinaison WIT/CIT est associée à la perte du greffon après la transplantation. Sachant qu’il s’agit de variables distinctes avec des déterminants différents, nous soulignons l’importance de rapporter la WIT et la CIT de façon indépendante. Qui plus est, les efforts visant à réduire la WIT et la CIT devraient être prioritaires.

## Introduction

Kidney transplantation is the optimal form of kidney replacement therapy and is associated with improved survival and quality of life compared with dialysis. Therefore, improving kidney organ survival after transplantation is of paramount importance. Minimizing both warm and cold ischemic injury is an areas of focus for the international transplant community.^1-6^ Studies have shown that warm ischemia time (WIT; typically defined as removal of an organ from cold storage to completion of reperfusion of warm blood following anastomosis)^3,7^ and cold ischemia time (CIT; the duration of time an organ spends in cold storage prior to transplantation)^7^ are important determinants of kidney allograft outcomes.^2^ Both prolonged WIT and CIT have been shown to independently impact kidney allograft survival. Each additional hour of CIT increases the risk of graft failure and mortality.^2^ In addition, we previously showed that prolonged WIT is associated with delayed graft function (DGF), graft failure and death after transplantation.^3^

Although WIT is a well-known risk factor for patient and organ survival after transplantation, as of 2015, WIT is not a mandatory variable that is collected in the United States Organ Network (UNOS) database. Furthermore, systematic capture of WIT is not prioritized in other transplant registries.^8^ Changes to organ allocation and distribution (as evident in the United States) may lead to longer CIT in both live and deceased donor recipients.^9,10^ Therefore, having a better understanding of the impact of WIT and CIT on post-transplant outcomes is critical, as both of these are potentially modifiable.^11-14^

Therefore, the objective of this study was to examine the combined impact of WIT and CIT on (1) death or graft failure and (2) DGF in a cohort of kidney transplant recipients. We hypothesized that combined prolonged warm and cold ischemia would be associated with a higher risk of all-cause graft failure and a higher risk of DGF following kidney transplantation.

## Methods

### Study Design

We analyzed a national cohort of kidney transplant recipients from January 2000 to March 2015. Patients were followed from the date of transplantation to death, graft failure, end of follow-up, or the last date of follow-up (September 1, 2017). The Scientific Registry of Transplant Recipients (SRTR) was used to identify patients who underwent renal transplantation during the period of follow-up. The SRTR data system includes data on all donors, waitlisted candidates, and transplant recipients in the United States, submitted by the members of the Organ Procurement and Transplant Network (OPTN). The Health Resources and Services Administration, US Department of Health and Human Services, provides oversight to the activities of the OPTN and SRTR contractors. We included only those with complete data for WIT and CIT and excluded patients under 18, those who received multiple organs, or those who received sequential transplants as has been done in previous studies exploring the independent impact of WIT and CIT on outcomes.^2-5,15,16^ The article was prepared according to Strengthening the Reporting of Observational Studies in Epidemiology (STROBE) reporting guidelines (Supplemental Table S1).^17^

### Exposure Definition

Our primary exposure variable was derived from the combination of WIT and CIT. Although both variables were included in the SRTR during the time period of cohort inclusion, WIT was no longer separately reported as of end of March 2015; rather, it was combined with CIT and reported as a single value.^18,19^ As per the SRTR, CIT was defined as the number of hours that the kidney organ was in cold storage beginning from donor kidney cross-clamp.^7^ Warm ischemia time was defined as the time in minutes from removal of the kidney from cold storage to reperfusion with warm blood (venous or arterial), inclusive of surgical anastomosis time.

For both WIT and CIT, outliers and potentially erroneous values were removed as has been done in prior literature. Specifically, WITs of less than 10 minutes and greater than 120 minutes were excluded from the analysis due to the possibility of data entry errors or implausible values.^3^ For deceased donor recipients, CITs of less than 1 hour and greater than 48 hours were removed, and for live donor recipients, CITs of 0 hours and greater than 24 hours were removed. Subsequent to data exclusions, to create a composite variable that combined both WIT and CIT, CIT and WIT were categorized primarily using a data-driven approach. For the data-driven approach, we used cubic splines that have been previously used to categorize continuous predictors in multivariable modeling.^20^ Cubic splines produce a series of knots, or end points, that best approximate a function. This method was chosen over other categorization methods, such as quartiles, as it more optimally categorizes continuous data. For deceased donor recipients, CIT was categorized as 1 to <7.75, 7.75 to <16.43, 16.43 to <28, 28 to ≤48 hours and WIT as 10 to <25, 25 to <38, 38 to <63, and 63 to ≤120 minutes. For living donor recipients, CIT was categorized as >0 to <0.42, 0.42 to <1, 1 to <3.04, 3.04 to ≤24 hours and WIT as 10 to <23, 23 to <35, 35 to <60, 60 to ≤120 minutes. This led to a single 16-level categorical variable combining both WIT and CIT separately for each of live and deceased donor recipients.

#### Covariates/confounders

Potential confounders of interest were collected from the SRTR database on the basis of their perceived association with the exposure (WIT/CIT) and outcomes of interest. These included recipient characteristics (age, sex, race, body mass index, dialysis vintage, cause of end-stage kidney disease, and presence of comorbidities: diabetes mellitus, hypertension, coronary artery disease or angina, peripheral vascular disease, cerebrovascular disease, prior malignancy, and prior kidney transplant), donor characteristics (age, sex, race, body mass index, history of hypertension, hepatitis C status, and history of diabetes mellitus), transplant/surgical characteristics (donor transplant side and expanded criteria donor and donation after circulatory death for deceased donors), and immunologic characteristics (human leukocyte antigen mismatch and peak panel reactive antibody).^3,21^

#### Outcome

The primary outcome of interest was a composite of death and graft failure (all-cause graft failure). Graft failure was defined as a permanent return to dialysis or pre-emptive retransplantation. In a prespecified secondary analysis, we analyzed each component of the primary outcome separately (ie, death-censored graft failure and death with graft function). Delayed graft function was also evaluated as a secondary outcome and defined as the need for dialysis in the first week post-transplantation.

#### Analysis

Baseline characteristics were reported separately for live and deceased donor kidney transplant recipients. For all baseline characteristics, categorical variables were reported as counts and percentages and continuous variables were displayed as means and standard deviations or medians with Q1-Q3 for non-normally distributed continuous variables. The WIT and CIT for live and deceased donors were graphically displayed using histograms. Time to all-cause graft failure for each category of WIT/CIT was analyzed using multivariable Cox proportional hazard models; the association between WIT/CIT and graft failure was reported using relative hazard ratio (HR) with 95% confidence intervals (CIs). For all models, the reference group was the shortest combination of WIT/CIT. Missingness was handled using case-wise deletion. For all Cox models, proportionality was assessed using visual examination of log-log plots. For the primary analysis, we adjusted for donor factors, recipient factors, and surgical and immunologic factors noted above. For DGF, we used logistic regression and reported the odds ratios (ORs) and 95% CIs for each category of WIT/CIT, adjusting for the same variables. In addition to the primary analysis, we performed 4 additional sensitivity analyses to test the robustness of our primary finding. (1) the effect of combined WIT/CIT on the primary outcome was analyzed using a clinical approach by categorizing CIT and WIT into short, medium, and long durations using thresholds defined by the available literature.^2,3,15,21^ In this analysis, for recipients of deceased and living donors, WIT was divided into categories of 10 to <30, 30 to <60, and 60 to ≤120 minutes.^4^ As CIT is generally longer for deceased donor recipients, CIT for deceased donors was categorized as 1 to <12, 12 to <24, and 24 to ≤48 hours and for living donors as >0 to <1, 1 to <3, and 3 to ≤24 hours. (2) The impact of combined WIT/CIT on the primary and secondary outcome (DGF) was analyzed inclusive of all values of WIT/CIT (including extreme values). (3) To remove the potential impact of prolonged ischemia time during the DCD withdrawal process (which was not robustly available in this data set), the impact of combined WIT/CIT on the primary outcome was analyzed among deceased donor recipients but excluding DCD recipients. (4) To address missing data and acknowledging the primary analytic approach used case-wise deletion, the primary analysis was repeated using multiple imputation with chained equations. Variables used to impute missing values included donor characteristics: race, body mass index (BMI), history of diabetes mellitus, hepatitis C positive, hypertension; recipient characteristics: sex, race, BMI, previous kidney transplant, cause of end-stage renal disease, dialysis vintage; and immunologic characteristics: number of human leukocyte antigen (HLA) mismatches, panel-reactive antibody (PRA) category. The impact of combined WIT/CIT on all-cause graft failure was evaluated with the data set following the imputation. In addition to sensitivity analyses, the impact of combined WIT/CIT on all-cause graft failure was evaluated across prespecified subgroups of interest including expanded versus standard criteria donor status (deceased donor recipients only),^22^ donation after circulatory death (deceased donor recipients only), age (using a threshold of ≥65), sensitization (using a panel reactive antibody threshold of >80), and elevated BMI (using a threshold of >35 kg/m^2^). For subgroup analyses, combined WIT/CIT was treated as a binary variable (longest WIT/CIT vs all other categories) and separately analyzed for each of live and deceased donor recipients (using the data driven approach). All statistical analyses were performed using Stata version 16.1 (Stata Corp., College Station, Texas).

## Results

**Figure 1.**
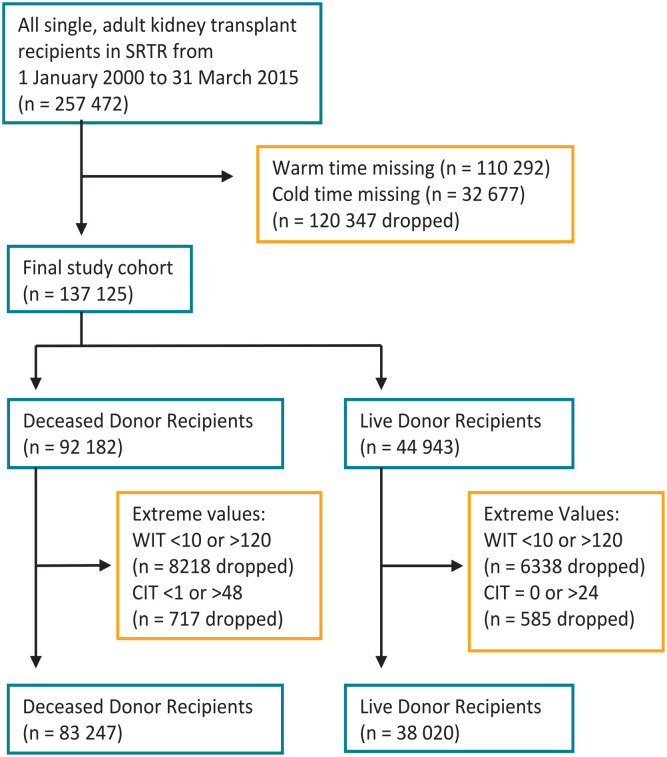
Derivation of study cohort. SRTR = Scientific Registry of Transplant Recipients; CIT = cold ischemia time (hours); WIT = warm ischemia time (minutes).

The final study cohort included 137 125 patients, consisting of 83 247 (61%) deceased donor recipients and 38 020 (39%) live donor recipients (Figure 1). Baseline characteristics for deceased donor recipients and live donor recipients are reported in Supplemental Table S2.

For live donor recipients, median (interquartile range [IQR]) WIT was 33 (25-45) minutes and median (IQR) CIT was 1 (0.95 to 2) hours (Supplemental Table S2). For deceased donor recipients, median (IQR) WIT was 36 (29-47) minutes and median (IQR) CIT was 17 (11.5-23) hours (Table 1). Distributions of WIT and CIT (using the final study cohort after exclusions) are noted in Supplemental Figure S1. The number of patients in each category of WIT, CIT, and combined WIT/CIT (for live and deceased donors using the data-driven and clinical approach) is noted in Table 1. For deceased donor recipients using the data-driven category, there were 8416 patients (10.1%) with prolonged WIT (63 to ≤120 minutes) and 8895 (10.7%) patients with prolonged CIT (28 to ≤48 hours) (Table 1). Similarly, for living donor recipients within the data-driven category, 3842 (4.6%) had prolonged WIT (60 to ≤120 minutes) and 3806 (10.0%) patients had prolonged CIT (3.04 to ≤24 hours) (Table 1).

**Table 1.** Number and Proportion of Patients in Each Category of WIT, CIT, and Combined WIT/CIT (Data-Driven and Clinically Driven Approach).

Data-driven approach					
*Deceased donor recipients (N=83 247)*					
	Cold ischemia time categories (hours):	1 to <7.75 N = 8289 (10.0%)	7.75 to <16.43 N = 33 325 (40.0%)	16.43 to <28 N = 32 738 (39.3%)	28 to ≤48 N = 8895 (10.7%)
Warm ischemia time categories (min):	10 to <25 N = 7842 (9.4%)	N = 1061	N = 3359	N = 2807	N = 615
	25 to <38 N = 32 593 (39.2%)	N = 3600	N = 13 382	N = 12 657	N = 2954
	38 to <63 N = 34 396 (41.3%)	N = 2973	N = 13 305	N = 13 853	N = 4265
	63 to ≤120 N = 8416 (10.1%)	N = 655	N = 3279	N = 3421	N = 1061
*Live donor recipients (N=38 020)*					
	Cold ischemia time categories (h):	>0 to <0.42 N = 3689 (9.7%)	0.42 to <1 N = 7649 (20.1%)	1 to <3.04 N = 22 876 (60.2%)	3.04 to ≤24 N =3806 (10.0%)
Warm ischemia time categories (min):	10 to <23 N = 3411 (4.1%)	N = 613	N = 854	N = 1650	N = 294
	23 to <35 N = 14 073 (16.9%)	N = 1850	N = 3368	N = 7595	N = 1260
	35 to <60 N = 16 694 (20.1%)	N = 1011	N = 3025	N = 10 911	N = 1747
	60 to ≤120 N = 3842 (4.6%)	N = 215	N = 402	N = 2720	N = 505
Clinical approach					
*Deceased donor recipients (N=83 247)*					
	Cold ischemia time categories (h):	1 to <12 N = 22 465 (27.0%)	12 to <24 N = 43 780 (52.6%)	24 to ≤48 N = 17 002 (20.4%)	
Warm ischemia time categories (min):	10 to <30 N = 17 068 (20.5%)	N = 5512	N = 8806	N = 2750	N/A
	30 to <60 N = 55 008 (66.1%)	N = 14 352	N = 29 024	N = 11 632	N/A
	60 to ≤120 N = 11 171 (13.4%)	N = 2601	N = 5950	N = 2620	N/A
*Live donor recipients (N* = *38 020)*					
	Cold ischemia time categories (hours):	>0 to <1 N = 11 338 (29.8%)	1 to <2 N = 15 295 (40.2%)	2 to ≤24 N = 11 387 (30.0%)	
Warm ischemia time categories (min):	10 to <30 N = 10 014 (12.0%)	N = 4163	N = 3608	N = 2243	N/A
	30 to <60 N = 24 164 (29.0%)	N = 6558	N = 9842	N = 7764	N/A
	60 to ≤120 N = 3842 (4.6%)	N = 617	N = 1845	N = 1380	N/A

### Primary Outcome (Death and Graft Failure)

**Figure 2.**
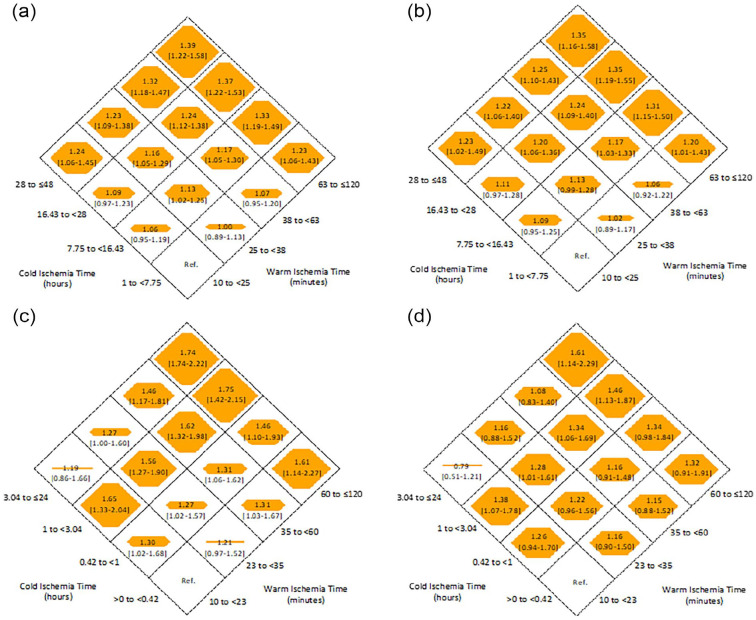
Relative hazard (with 95% confidence intervals) of all-cause graft failure and each category of warm and cold ischemia time among (a) deceased donor recipients (unadjusted model), (b) deceased donor recipients (adjusted model), (c) live donor recipients (unadjusted model), and (d) live donor recipients (adjusted model).

Among deceased and live donor recipients, there were a total of 38 755 and 13 113 primary events, respectively. Overall, cumulative time at risk was 784 631 patient years (504 013 for deceased donor recipients and 280 618 for live donor recipients), and median follow-up time was 5.74 (3.14-8.80) and 4.90 (2.86-7.85) for deceased and live donor recipients, respectively. For deceased donor recipients, compared with the reference category (WIT 10 to <25 minutes, CIT 1 to <7.75 hours), those with both prolonged WIT (63 to ≤120 mins) and prolonged CIT (28 to ≤48 hours) had a 35% increase in the adjusted relative hazard for all-cause graft loss (HR = 1.35, 95% CI = 1.16-1.58; Figure 2b and Supplemental Table S3). There was a graded increase in the point estimate for graft failure with each increase in prolonged WIT/CIT, but confidence intervals overlapped. Among live donor recipients, the risk of death/graft failure was 61% higher for those with a WIT of 60 to ≤120 minutes and CIT of 3.04 to ≤24 hours (HR = 1.61, 95% CI = 1.14-2.29) compared with the reference category (Figure 2d and Supplemental Table S3). A graded increase in the point estimate for graft failure was noted for those in the highest category of WIT with each increase in CIT, although confidence intervals overlapped. The impact of combined WIT/CIT on each subcomponent of the primary analysis is shown in Supplemental Table S4. Among deceased donor recipients, the highest point estimate for death with graft function was found in those with prolonged WIT/CIT. Among live donor recipients, the findings were inconsistent for both death with graft function and death-censored graft failure.

### Secondary Outcomes (DGF)

**Figure 3.**
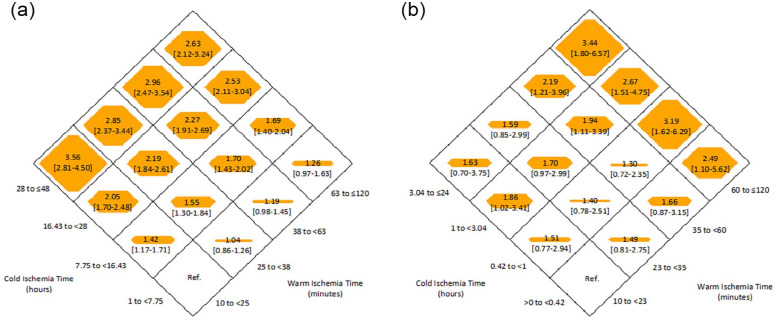
Adjusted odds ratio (with 95% confidence intervals) of delayed graft failure for each category of warm and cold ischemia among (a) deceased donor recipients and (b) live donor recipients.

The risk of DGF for combined WIT/CIT is noted in Figure 3 and Supplementary Table S5. For deceased donor recipients, the risk of DGF was highest for prolonged CIT (28 to ≤48 hours), irrespective of the WIT; the highest risk (OR = 3.56, 95% CI = 2.81-4.50) was observed for those with combined short WIT (10 to <25 minutes) and long CIT (28 to ≤48 hours). For live donor recipients, the relationship between prolonged WIT/CIT was not as consistent; however, the highest risk (OR = 3.44, 95% CI = 1.80-6.57) was observed among those with the longest WIT/CIT (WIT 60 to ≤120 minutes and CIT 3.04 to ≤24 hours).

### Sensitivity Analyses

The results were generally similar using clinical categories to define combined WIT/CIT (Figures 2a and c, and Supplemental Table S6). Among deceased donor recipients, the adjusted HR for death/graft failure for a WIT of 60 to ≤120 minutes and CIT of 24 to ≤48 hours was 1.26 (95% CI = 1.17-1.37). Among live donor recipients, the risk of graft loss for combined WIT/CIT was only significant for those in the highest category of WIT (60 to ≤120 minutes) and CIT (HR = 1.21, 95% CI = 1.01-1.46). When including all values of WIT/CIT (without exclusion of extraneous values), results were similar for those with prolonged WIT/CIT; however, the risk was also high for some categories of short WIT (Supplemental Table S7). The impact of removing DCD recipients yielded similar results to the primary analysis, wherein a graded response was observed particularly among the longest categories of WIT/CIT (Supplemental Table S8). Similarly, the implementation of multiple imputation using chained equations provided similar results to the analysis using case-wise deletion (Supplemental Table S9), although the graded increase in risk of CIT for each incrementally higher WIT category was not as evident, particularly for live donor recipients using the data-driven approach.

### Subgroup Analyses

Among deceased donor recipients, there were no significant differences in the impact of prolonged WIT/CIT (63 to ≤120 minutes/28 to ≤48 hours) across subgroups of interest (Table 2). A potential association was observed among live donor recipients, wherein prolonged WIT/CIT (60 to ≤120 minutes/3.04 to ≤24 hours) was associated with death/graft loss in those with a PRA of ≥80 (HR = 2.55, 95% CI = 1.29-5.04) but not in those with a PRA of <80 (HR = 1.17 95% CI = 0.87-1.56, interaction *P* = .050, Table 2).

**Table 2.** Association Between Combined Long WIT/CIT and All-Cause Graft Failure Across Subgroups of Interest (Data-Driven Approach).

Model	Relative hazard, HR [95% CI]	Relative hazard, HR [95% CI]	Interaction
Deceased donor recipients			
Age	<65 y	≥65 y	
Patients/events	51 749/20 580	11 509/5952	
	1.45 [1.03-1.28]	1.08 [0.87-1.34]	*P* = .450
BMI	≤34.99	>34.99	
Patients/events	56 370/23 493	6888/3039	
	1.11 [1.00-1.23]	1.33 [1.04-1.69]	*P* = .237
ECD	Standard criteria donor	Expanded criteria donor	
Patients/events	42 045/16 976	7715/4481	
	1.10 [0.97-1.24]	1.42 [1.13-1.77]	*P* = .212
DCD	Not-DCD	DCD	
Patients/events	55 499/11 165	7759/1262	
	1.04 [0.90-1.21]	1.50 [0.87-2.60]	*P* = .280
PRA category	<80	≥80	
Patients/events	54 777/10 594	9914/1962	
	1.05 [0.90-1.23]	1.15 [0.81-1.63]	*P* = .514
Donor creatinine (mg/dL)	<2.26	≥2.26	
Patients/events	60 056/25 388	3173/1127	
	1.14 [1.03-1.26]	1.09 [0.72-1.64]	*P* = .903
Live donor recipients			
Age	<65 y	≥65 y	
Patients/events	19 937/4289	2808/955	
	1.21 [0.87-1.67]	1.42 [0.88-2.28]	*P* = .761
BMI	≤34.99	>34.99	
Patients/events	20 381/4646	2364/598	
	1.35 [1.02-1.80]	0.99 [0.43-2.28]	*P* = .412
PRA category	PRA <80	PRA ≥80	
Patients/Events	21 528/4913	2492/562	
	1.17 [0.87-1.56]	2.55 [1.29-5.04]	*P* = .050^a^

*Note.* Adjusted for donor characteristics: donor age, donor sex, donor race, donor body mass index category, donor hypertension, donor diabetes, donor creatinine, and donor hepatitis C status; donor type: expanded donor criteria, donation after circulatory death (for deceased donors only); immunologic characteristics: number of human leukocyte antigen mismatches, panel reactive antibody category; surgical characteristics: CIT, WIT, donor transplant side; and recipient characteristics: age, sex, recipient race, body mass index category, dialysis vintage, cause of end-stage renal disease, recipient diabetes, hypertension, coronary artery disease, cerebrovascular disease, peripheral vascular disease, prior malignancy, previous kidney transplant. BMI = body mass index; CI = confidence interval; DCD = donation after circulatory death; ECD = expanded donor criteria; HR = hazard ratio; PRA = panel reactive antibody; WIT = warm ischemia time; CIT = cold ischemia time.

a Significant *P* < 0.05.

## Discussion

In this study, we identified that prolonged WIT and CIT were associated with an increased risk of graft failure and death after transplantation. This association was largely consistent using two different approaches to creating a combined WIT/CIT variable, and although the magnitude of the effect differed and CIs overlapped, it was present among live and deceased donor recipients after adjustment for confounding variables.

Our results are consistent with previous work (including our own) indicating that separately considered, prolonged WIT and CIT are associated with adverse outcomes. Prolonged CIT has been shown to be an independent predictor of graft survival^23^ and a principal factor leading to DGF.^15,23^ It has previously been shown that each additional hour of CIT is associated with an increased risk of graft failure and mortality.^2^ Similarly, Hernandez et al demonstrated that each 5-hour increase of CIT was associated with a 20% increase of death-censored graft loss.^23^ In this study, we demonstrated an association with both graft loss and DGF. Regarding DGF, among deceased donors, the effect was more related to CIT wherein WIT showed minimal impact on DGF when CIT was prolonged. The highest point estimates for DGF were noted even in those with short WIT, suggesting that the mechanisms causing DGF may occur prior to WIT. It is for reasons like these that we emphasize the importance of capturing WIT independently of CIT.

Our previous work with data from the United States demonstrated that prolonged WIT was associated with an increased relative hazard of death and graft failure.^3,24^ The impact of prolonged WIT on long-term graft survival has also been shown in cohorts outside of the United States, that is, in both Japan and the Netherlands.^4,25^ Irish and colleagues were among the first to identify the impact of CIT on DGF, although they did not include WIT in their risk prediction nomogram.^26^ Although these relationships have been established, our study is the first, to our knowledge, to examine the combined impact of WIT and CIT on both surrogate (DGF) and end outcomes of death and graft failure, using both a data-driven approach and clinical approach based on literature-informed thresholds.

It has been shown that prolonged CIT is hazardous to long-term graft survival^27^; however, the exact mechanisms responsible for this damage are still not fully understood. Some suggested mechanisms for cold ischemia include hypoperfusion, induced hypoxic injuries, and ultimately ischemia-reperfusion injury,^6,13^ whereas for warm ischemia, hemodynamic stress, cellular injury, and activation of inflammatory mediators have all been implicated.^28,29^ While the reason underpinning the higher combined effect of both WIT and CIT remains to be determined, we speculate that the combination of prolonged WIT/CIT results in a “two-hit” model of injury to the graft, wherein grafts exposed to both prolonged WIT and CIT time may be more susceptible due to cumulative damage through multiple pathways. Beyond the pathophysiological impact, we must also consider that the impact of prolonged WIT/CIT may be secondary to additional factors not captured within the data set. These factors may include quality of the graft, recipient anatomy or body habitus, or use of ischemia-reducing strategies such as cooling blankets, perfusion machines (hypothermic machine), or other innovative solutions.^30^ Nonetheless, the impact of combined WIT/CIT seemed to persist even for live donor recipients, suggesting that this risk is present even in higher quality and more resilient organs.

One interesting finding in this study was that there was an association between increasing WIT/CIT and the outcome of death with graft function. We consider many possible reasons for this observation. First, as chronic kidney disease is known to be independently associated with mortality risk, the impact of WIT/CIT on kidney function, even to a small degree, may pose downstream effects on survival. Second, both WIT and CIT have been shown to induce inflammation,^1,31^ which can impact patient complications and subsequently lead to increased mortality risk.^32^ Finally, there are unmeasured recipient characteristics that may prolong CIT and WIT, such as the degree of frailty or additional comorbidities, which may contribute to a heightened mortality risk. For example, recipient frailty may influence donor selection, leading to prolonged CIT, or may lead to prolonged WIT as a result of increased surgical time in the setting of a patient with underlying comorbidities or health deficits.

While finding ways to reduce CIT seems intuitive, due to the nature of the solid organ sharing program in the United States, shortening CIT may not be possible, especially if the focus is on maintaining equitable access to organs.^33-35^

At present, the Organ Procurement and Transplantation Network (OPTN) is responsible for organ allocation in the United States. The kidney allocation system considers several factors such as the calculated panel reactive antibody (CPRA), estimated post-transplant survival (EPTS) score, HLA and ABDR mismatches, recipient waiting time, and distance within the donation service area (DSA).^35^ If the DSA is quite large, it may be impossible to reduce CIT times. Our previous work has demonstrated that efforts to reduce HLA mismatches are important, but the benefits are not as clear if the duration of CIT exceeds 20 hours.^16^ As such, CIT duration should be a consideration when transporting organs across large DSAs. Furthermore, efforts to minimize cold time with hypothermic machine perfusion have been shown to reduce the rates of DGF (from deceased donors) and improve short-term outcomes.^36,37^ If CIT is not easily modifiable, identifying novel ways to reduce WIT (especially for those at highest risk due to prolonged CIT) may be another consideration. These innovative approaches include minimizing rewarming during anastomosis through devices such as cooling jackets or devices that prevent rewarming of the kidney during anastomosis.^12^ Although WIT may be shortened by reducing anastomosis time, it is likely that longer anastomosis times (especially in the face of anastomotic anomalies) may not be avoidable. Similar to CIT, there are unmodifiable factors associated with prolonged WIT, including recipient BMI and kidney type (ie, right donor kidney).^21^ Furthermore, as suggested by prior literature, speeding up the time of surgical anastomosis may also be associated with additional risk.^11^

Of importance, prolonged WIT/CIT (albeit using different thresholds) was still associated with a higher relative hazard of all-cause graft loss among live donor recipients. It is acknowledged that CIT is typically shorter in live donor recipients. While CIT may not be equitably modified for deceased donor recipients, concerted efforts to keep CIT short for live donor recipients would be supported by the results of our study. It should be emphasized though that the impact of prolonged CIT on the composite outcome was only observed among those with the longest WIT, something that cannot necessarily be anticipated in advance.

While the impact of WIT/CIT on graft failure was not modified by a number of different factors, we did identify that patients who were highly sensitized and had prolonged WIT/CIT were at a very high risk of graft failure with an adjusted relative hazard of 2.55 (Table 2). It has previously been shown that highly sensitized patients who also experience DGF may be at an even higher risk of acute rejection (although a link with graft loss was not found in this study).^38^ In addition, it has been shown that DGF was reduced particularly among highly sensitized patients with shorter CIT.^39^ In another study, optimizing the timing of pretransplant HLA testing leads to a reduction in CIT, and the effect on DGF was more pronounced among those who were highly sensitized (PRA >80%).^5^ Thus, a more detailed analysis of the impact on prolonged WIT/CIT among sensitized patients would be of interest for future study, and sensitized patients may be most likely to benefit from concerted efforts to lower CIT/WIT, appreciating that ensuring access to organs for sensitized patients through national sharing may limit the ability to shorten CIT without reducing the donor pool for a sensitized recipient. However, it is important to appreciate that the interaction *P* value was only borderline significant, and thus there was a risk of a type 1 error (false positive rate). Therefore, further analysis into the impact of sensitization and intersection of prolonged WIT/CIT and development of donor specific antibodies would be of interest for future study.

Our study had many strengths. The size of the data set allowed for adjustment of many donor and recipient factors and improved generalizability. Furthermore, we demonstrated robustness of our findings through consistent results using 2 different approaches to categorizing WIT/CIT, sensitivity analyses, and key subgroups of interest. However, there were limitations to our study. First, in this administrative data set, we did not have access to some aspects of the transplantation. For example, the data set did not contain information surrounding donor warm time during organ retrieval or direct temperature assessments of the kidney. These datapoints would have been of value to our analyses. Recordings of WIT can be unreliable and a surrogate marker such as direct temperature assessment would have been valuable to complement WIT. In addition, we recognize that WIT may be impacted by dynamic elements surrounding the withdrawal for DCD donors which were unavailable within the data set. As usage of DCD kidneys continues to increase,^40^ the concept of functional WIT and nuances of data recording remain important considerations for future contemporary work. Moreover, data for transplantation surgical complexity, the presence of learners, skill of the surgeon, and use of additional devices or technology were not recorded. These factors can impact the duration of surgery and may contribute to outcomes. Second, the dataset contained variables with considerable missingness, such as whether kidneys were placed on pump perfusion, which were not robust enough to include in our analyses. Usage of pump perfusion as an organ preservation technique has become widespread, but accurate data on the use of these pumps were not available in our study. Kidneys that have been placed on pump perfusion have been shown to reduce DGF and as such would have been an important contribution to our study.^37^ Third, there is a question surrounding miscoded or erroneous values especially at extreme values. To account for this, we removed very short and very long times in our primary analysis, leading to the exclusion of a large number of live and deceased donor recipients. While this was a limitation, we did demonstrate similar results when all data were included. We do acknowledge that while point estimates suggested the highest risk of death/graft failure for combined WIT/CIT, CIs were wide and overlapping. Therefore, it is possible that statistically significant differences in the incremental increase in risk would not have been observed in an even larger cohort. Finally, we acknowledge a potential reporting bias due to missing WIT and CIT as well as the potential for unmeasured confounding variables that may alter conclusions.

## Conclusions

In conclusion, we demonstrated that combined WIT/CIT is associated with all-cause graft failure and DGF following kidney transplantation. Acknowledging that both WIT and CIT are potentially modifiable, efforts should be made to identify those at highest risk, to reduce WIT where feasible, and to reduce CIT (without compromising efforts at equitable distribution of organs). Strategies to reduce WIT/CIT or reduce the consequences of injury in those with prolonged WIT/CIT should continue to be prioritized for future research.

## Supplemental Material

sj-docx-1-cjk-10.1177_20543581231178960 – Supplemental material for The Impact of Combined Warm and Cold Ischemia Time on Post-transplant Outcomes Click here for additional data file. Supplemental material, sj-docx-1-cjk-10.1177_20543581231178960 for The Impact of Combined Warm and Cold Ischemia Time on Post-transplant Outcomes by Martha E. Foley, Amanda J. Vinson, Thomas A. A. Skinner, Bryce A. Kiberd and Karthik K. Tennankore in Canadian Journal of Kidney Health and Disease
