# Supplementary material for: The Impact of Combined Warm and Cold Ischemia Time on Post-transplant Outcomes
Source: Can J Kidney Health Dis. 2023 Jun 11;10:20543581231178960. doi: 10.1177/20543581231178960 (PMC10272701; doi:10.1177/20543581231178960)
Supplement: sj-docx-1-cjk-10.1177_20543581231178960 – Supplemental material for The Impact of Combined Warm and Cold Ischemia Time on Post-transplant Outcomes [file sj-docx-1-cjk-10.1177_20543581231178960.docx]

**Supplemental Table 1.** STROBE statement for cohort studies

|  | Item No | Recommendation | Page No |
| --- | --- | --- | --- |
| **Title and abstract** | 1 | (*a*) Indicate the study’s design with a commonly used term in the title or the abstract | 2 |
|  |  | (*b*) Provide in the abstract an informative and balanced summary of what was done and what was found | 2 |
| Introduction | | | |
| Background/rationale | 2 | Explain the scientific background and rationale for the investigation being reported | 3 |
| Objectives | 3 | State specific objectives, including any prespecified hypotheses | 3 |
| Methods | | | |
| Study design | 4 | Present key elements of study design early in the paper | 4 |
| Setting | 5 | Describe the setting, locations, and relevant dates, including periods of recruitment, exposure, follow-up, and data collection | 4 |
| Participants | 6 | (*a*) Give the eligibility criteria, and the sources and methods of selection of participants. Describe methods of follow-up | 4 |
|  |  | (*b*) For matched studies, give matching criteria and number of exposed and unexposed | n/a |
| Variables | 7 | Clearly define all outcomes, exposures, predictors, potential confounders, and effect modifiers. Give diagnostic criteria, if applicable | 4,5 |
| Data sources/ measurement | 8* | For each variable of interest, give sources of data and details of methods of assessment (measurement). Describe comparability of assessment methods if there is more than one group | 4 |
| Bias | 9 | Describe any efforts to address potential sources of bias | 6 |
| Study size | 10 | Explain how the study size was arrived at | 4 |
| Quantitative variables | 11 | Explain how quantitative variables were handled in the analyses. If applicable, describe which groupings were chosen and why | 5 |
| Statistical methods | 12 | (*a*) Describe all statistical methods, including those used to control for confounding | 6,7 |
|  |  | (*b*) Describe any methods used to examine subgroups and interactions | 6,7 |
|  |  | (*c*) Explain how missing data were addressed | 6 |
|  |  | (*d*) If applicable, explain how loss to follow-up was addressed | n/a |
|  |  | (*e*) Describe any sensitivity analyses |  |
| Results | | |  |
| Participants | 13* | (a) Report numbers of individuals at each stage of study—eg numbers potentially eligible, examined for eligibility, confirmed eligible, included in the study, completing follow-up, and analysed | 7 |
|  |  | (b) Give reasons for non-participation at each stage | Fig 1 |
|  |  | (c) Consider use of a flow diagram | Fig 1 |
| Descriptive data | 14* | (a) Give characteristics of study participants (eg demographic, clinical, social) and information on exposures and potential confounders | Table S2 |
|  |  | (b) Indicate number of participants with missing data for each variable of interest | Table S2 |
|  |  | (c) Summarise follow-up time (eg, average and total amount) | 9 |
| Outcome data | 15* | Report numbers of outcome events or summary measures over time | 9 |

| Main results | 16 | (*a*) Give unadjusted estimates and, if applicable, confounder-adjusted estimates and their precision (eg, 95% confidence interval). Make clear which confounders were adjusted for and why they were included | 9,10  Figures 2,3 |
| --- | --- | --- | --- |
|  |  | (*b*) Report category boundaries when continuous variables were categorized | 7 |
|  |  | (*c*) If relevant, consider translating estimates of relative risk into absolute risk for a meaningful time period | n/a |
| Other analyses | 17 | Report other analyses done—eg analyses of subgroups and interactions, and sensitivity analyses | 10,11 |
| Discussion | | | |
| Key results | 18 | Summarise key results with reference to study objectives | 12 |
| Limitations | 19 | Discuss limitations of the study, taking into account sources of potential bias or imprecision. Discuss both direction and magnitude of any potential bias | 16 |
| Interpretation | 20 | Give a cautious overall interpretation of results considering objectives, limitations, multiplicity of analyses, results from similar studies, and other relevant evidence | 14-17 |
| Generalisability | 21 | Discuss the generalisability (external validity) of the study results | 16 |
| Other information | | | |
| Funding | 22 | Give the source of funding and the role of the funders for the present study and, if applicable, for the original study on which the present article is based | 17 |

**Supplemental Table S2.** Baseline characteristics after exclusions and extraneous values, by donor type

| **Baseline Characteristics**  **[n(%)] unless stated** | **Eligible study cohort**  **(N = 137 125)** | **Proportion with missing data** | **Deceased Donor Recipients**  **(N = 92 182)** | **Live Donor**  **Recipients**  **(N = 44 943)** |
| --- | --- | --- | --- | --- |
| **Donor Characteristics** |  |  |  |  |
| Age (mean ±SD) | 39.5(±14.6) | <1% | 38.4 (± 15.8) | 41.4 (±11.5) |
| Male sex | 73 154 |  | 55 370 (60.1%) | 17 784 (39.6%) |
| *Race* |  |  |  |  |
| White | 113 972 | <1% | 56 309 (61.1%) | 35 954 (80.0%) |
| Black | 18 191 |  | 28 941 (31.4%) | 6 532 (14.5%) |
| Other | 4 947 |  | 6 932 (7.5%) | 2 453 (5.5%) |
| *BMI Categories, kg/m^2^* |  | 1.4% |  |  |
| <18.49 | 5 262 |  | 2 122 (2.3%) | 1 264 (2.8%) |
| >18.49-24.99 | 48 688 |  | 26 402 (28.6%) | 14 441 (34.1%) |
| >24.99-29.99 | 46 810 |  | 29 390 (31.9%) | 14 013 (31.2%) |
| >29.99-34.99 | 22 682 |  | 19 233 (20.9%) | 8 692 (19.3%) |
| >34.99 | 11 722 |  | 9 431 (10.2%) | 4 103 (9.1%) |
|  |  |  |  |  |
| **Transplant or Surgical Characteristics** |  |  |  |  |
| WIT, minutes, median | 35 (27-45) |  | 36 (29-47) | 33 (25-45) |
| CIT, hours, median | 11.8 (2-19.5) |  | 16.5 (11.2-22.4) | 1 (0.85-2) |
|  |  |  |  |  |
| Expanded Criteria Donor | 14 471 | **---** | 14 471 (15.7%) | n/a |
| Donation after circulatory death | 10 883 | <1%**^a^** | 10 883 (11.8%) | n/a |
| Donor hepatitis C positive | 2 103 | <1%**^a^** | 2 103 (2.3%) | n/a |
| History of diabetes mellitus | 5 910 | <1%**^a^** | 5 910 (6.4%) | n/a |
| Hypertension | 24 645 | 6.7% | 23 722 (25.7%) | 923 (2.1%) |
| Transplant side (right) | 53 697 |  | 48 137 (52.2%) | 5 560 (12.4%) |
|  |  |  |  |  |
| **Immunologic Characteristics** |  |  |  |  |
| *HLA mismatches* |  | <1% |  |  |
| 0 | 12 235 |  | 8 568 (9.3%) | 3 667 (8.2%) |
| 1 | 4 792 |  | 2 483 (2.7%) | 2 311 (5.1%) |
| 2 | 11 502 |  | 4 283 (4.6%) | 7 219 (16.1%) |
| 3 | 23 877 |  | 11 990 (13.0%) | 11 887 (26.4%) |
| 4 | 30 165 |  | 23 246 (25.2%) | 6 919 (15.4%) |
| 5 | 35 478 |  | 27 338 (29.7%) | 8 140 (18.1%) |
| 6 | 18 061 |  | 13 708 (14.9%) | 4 353 (9.7%) |
|  |  |  |  |  |
| PRA category |  | 3.3% |  |  |
| 0-<20 | 94 881 |  | 60 519 (65.7%) | 34 362 (76.5%) |
| 20-80 | 23 814 |  | 17 355 (18.8%) | 6 459 (14.4%) |
| ≥80 | 13 847 |  | 11 774 (12.8%) | 2 073 (4.6%) |
|  |  |  |  |  |
|  |  |  |  |  |
| **Recipient Characteristics** |  |  |  |  |
| Age (mean ±SD) | 50.6 (±13.6) |  | 52.1 (±13.1) | 47.4 (±14.1) |
| Male sex | 83 283 |  | 56 060 (60.8%) | 27 223 (60.6%) |
| *Race* |  | <1% |  |  |
| White | 92 263 |  | 56 309 (61.1%) | 35 954 (80.0%) |
| Black | 35 475 |  | 28 941 (31.4%) | 6 534 (14.5%) |
| Other | 9 385 |  | 6 932 (7.5%) | 2 453 (5.5%) |
| *BMI Categories, kg/m^2^* |  | 5.8% |  |  |
| <=18.49 | 3 386 |  | 2 122 (2.3%) | 1 264 (2.8%) |
| >18.49-24.99 | 40 843 |  | 26 402 (28.6%) | 14 441 (32.1%) |
| >24.99-29.99 | 43 403 |  | 29 390 (31.9%) | 14 013 (31.2%) |
| >29.99-34.99 | 27 925 |  | 19 233 (20.9%) | 8 692 (19.3%) |
| >34.99 | 13 534 |  | 9 431 (10.2%) | 4 103 (9.1%) |
| *Dialysis vintage* |  | <1% |  |  |
| 0-0.5 year | 55 514 |  | 28 467 (30.9%) | 27 047 (60.2%) |
| 0.5-1 year | 6 807 |  | 2 796 (3.0%) | 4 011 (8.9%) |
| 1-2 years | 14 859 |  | 8 744 (9.5%) | 6 115 (13.6%) |
| 2-3 years | 14 263 |  | 10 981 (11.9%) | 3 282 (7.3%) |
| 3-4 years | 12 421 |  | 10 627 (11.5%) | 1 794 (4.0%) |
| >4 years | 33 259 |  | 30 565 (33.2%) | 2 694 (6.0%) |
| *Cause of ESKD* |  | 3.8% |  |  |
| Glomerulonephritis | 33 634 |  | 20 126 (21.8%) | 13 508 (30.1%) |
| Diabetes | 33 551 |  | 24 157 (26.2%) | 9 394 (20.9%) |
| Polycystic kidney disease | 13 103 |  | 7 921 (8.6%) | 5 182 (11.5%) |
| Hypertension | 32 278 |  | 24 199 (26.3%) | 8 079 (18.0%) |
| Other | 19 311 |  | 12 373 (13.4%) | 6 938 (15.4%) |
| *Comorbidities* |  |  |  |  |
| Diabetes mellitus | 42 970 | 1.1% | 31 056 (33.7%) | 11 914 (26.5%) |
| Hypertension | 112 743 | 5.8% | 76 262 (82.7%) | 36 481 (81.2%) |
| Coronary artery disease or angina | 10 964 | 10.0% | 7 958 (8.6%) | 3 006 (6.7%) |
| Peripheral vascular disease | 6 817 | 3.0% | 5 007 (5.4%) | 1 810 (4.0%) |
| Cerebrovascular disease | 3 547 | 8.4% | 2 630 (2.9%) | 917 (2.0%) |
| Prior malignancy | 7 881 | 2.5% | 5 204 (5.6%) | 2 677 (6.0%) |
| Prior kidney transplant | 16 277 | <1% | 11 711 (12.7%) | 4 566 (10.2%) |

BMI, body mass index; CIT, cold ischemia time; ESKD, end-stage kidney disease; HLA, human leukocyte antigen; PRA, panel reactive antibody; WIT, warm ischemia time

^a^Calculated only among deceased donor recipients

**Supplemental Table S3.** Association between combined WIT/CIT and all-cause graft failure

|  | **Warm Ischemia Time (minutes)** | **Cold Ischemia Time (hours)** | **Death or Graft Failure**  **Unadjusted HR [95%CI]** | **Death or Graft Failure**  **Adjusted HR [95%CI]** |
| --- | --- | --- | --- | --- |
| *Deceased Donor Recipients* |  |  |  |  |
|  | 10 to <25 | 1 to <7.75 | Ref. | Ref. |
|  | 10 to <25 | 7.75 to <16.43 | 1.06 [0.95-1.19] | 1.09 [0.95-1.25] |
|  | 10 to <25 | 16.43 to <28 | 1.09 [0.97-1.23] | 1.11 [0.97-1.28] |
|  | 10 to <25 | 28 to ≤48 | 1.24 [1.06-1.45] | 1.23 [1.02-1.49] |
|  | 25 to <38 | 1 to <7.75 | 1.00 [0.89-1.13] | 1.02 [0.89-1.17] |
|  | 25 to <38 | 7.75 to <16.43 | 1.13 [1.02-1.25] | 1.13 [0.99-1.28] |
|  | 25 to <38 | 16.43 to <28 | 1.16 [1.05-1.29] | 1.20 [1.06-1.36] |
|  | 25 to <38 | 28 to ≤48 | 1.23 [1.09-1.38] | 1.22 [1.06-1.40] |
|  | 38 to <63 | 1 to <7.75 | 1.07 [0.95-1.20] | 1.06 [0.92-1.22] |
|  | 38 to <63 | 7.75 to <16.43 | 1.17 [1.05-1.30] | 1.17 [1.03-1.33] |
|  | 38 to <63 | 16.43 to <28 | 1.24 [1.12-1.38] | 1.24 [1.09-1.40] |
|  | 38 to <63 | 28 to ≤48 | 1.32 [1.18-1.47] | 1.25 [1.10-1.43] |
|  | 63 to ≤120 | 1 to <7.75 | 1.23 [1.06-1.43] | 1.20 [1.01-1.43] |
|  | 63 to ≤120 | 7.75 to <16.43 | 1.33 [1.19-1.49] | 1.31 [1.15-1.50] |
|  | 63 to ≤120 | 16.43 to <28 | 1.37 [1.22-1.53] | 1.35 [1.19-1.55] |
|  | 63 to ≤120 | 28 to ≤48 | 1.39 [1.22-1.58] | 1.35 [1.16-1.58] |
| *Live-Donor Recipients* |  |  |  |  |
|  | 10 to <23 | >0 to <0.42 | Ref. | Ref. |
|  | 10 to <23 | 0.42 to <1 | 1.30 [1.02-1.68] | 1.26 [0.94-1.70] |
|  | 10 to <23 | 1 to <3.04 | 1.65 [1.33-2.04] | 1.38 [1.07-1.78] |
|  | 10 to <23 | 3.04 to ≤24 | 1.19 [0.86-1.66] | 0.79 [0.51-1.21] |
|  | 23 to <35 | >0 to <0.42 | 1.21 [0.97-1.52] | 1.16 [0.90-1.50] |
|  | 23 to <35 | 0.42 to <1 | 1.27 [1.02-1.57] | 1.22 [0.96-1.56] |
|  | 23 to <35 | 1 to <3.04 | 1.56 [1.27-1.90] | 1.28 [1.01-1.61] |
|  | 23 to <35 | 3.04 to ≤24 | 1.27 [1.00-1.60] | 1.16 [0.88-1.52] |
|  | 35 to <60 | >0 to <0.42 | 1.31 [1.03-1.67] | 1.15 [0.88-1.52] |
|  | 35 to <60 | 0.42 to <1 | 1.31 [1.06-1.62] | 1.16 [0.91-1.48] |
|  | 35 to <60 | 1 to <3.04 | 1.62 [1.32-1.98] | 1.34 [1.06-1.69] |
|  | 35 to <60 | 3.04 to ≤24 | 1.46 [1.17-1.81] | 1.08 [0.83-1.40] |
|  | 60 to ≤120 | >0 to <0.42 | 1.61 [1.14-2.27] | 1.32 [0.91-1.91] |
|  | 60 to ≤120 | 0.42 to <1 | 1.46 [1.10-1.93] | 1.34 [0.98-1.84] |
|  | 60 to ≤120 | 1 to <3.04 | 1.75 [1.42-2.15] | 1.46 [1.13-1.87] |
|  | 60 to ≤120 | 3.04 to ≤24 | 1.74 [1.37-2.22] | 1.61 [1.14-2.29] |

Adjusted for donor characteristics: donor age, donor sex, donor race, donor body mass index category, donor hypertension, donor diabetes, and donor hepatitis C status; donor type: expanded donor criteria, donation after circulatory death (for deceased donors only); immunologic characteristics: number of human leukocyte antigen mismatches, panel reactive antibody category; surgical characteristics: cold ischemia time, warm ischemia time, donor transplant side; and recipient characteristics: age, sex, recipient race, body mass index category, dialysis vintage, cause of end stage renal disease, recipient diabetes, hypertension, coronary artery disease, cerebrovascular disease, peripheral vascular disease, prior malignancy, previous kidney transplant.
CI, confidence interval; HR, hazard ratio

**Supplemental Table S4.** Adjusted association of WIT/CIT for component outcomes of graft loss and death

|  | **Warm Ischemia Time (minutes)** | **Cold Ischemia Time (hours)** | **Event:**  **Graft Loss**  **OR [95%CI]** | **Event:**  **Death^a^**  **OR [95%CI]** |
| --- | --- | --- | --- | --- |
| *Deceased Donor Recipients* |  |  |  |  |
|  | 10 to <25 | 1 to <7.75 | Ref. | Ref. |
|  | 10 to <25 | 7.75 to <16.43 | 1.02 [0.83-1.24] | 1.14 [0.94-1.37] |
|  | 10 to <25 | 16.43 to <28 | 1.09 [0.89-1.34] | 1.12 [0.93-1.36] |
|  | 10 to <25 | 28 to ≤48 | 1.16 [0.87-1.54] | 1.27 [0.98-1.65] |
|  | 25 to <38 | 1 to <7.75 | 1.03 [0.84-1.25] | 0.99 [0.81-1.20] |
|  | 25 to <38 | 7.75 to <16.43 | 1.08 [0.90-1.30] | 1.15 [0.97-1.37] |
|  | 25 to <38 | 16.43 to <28 | 1.17 [0.97-1.40] | 1.21 [1.02-1.45] |
|  | 25 to <38 | 28 to ≤48 | 1.18 [0.97-1.45] | 1.24 [1.02-1.50] |
|  | 38 to <63 | 1 to <7.75 | 1.01 [0.82-1.24] | 1.09 [0.90-1.32] |
|  | 38 to <63 | 7.75 to <16.43 | 1.14 [0.95-1.37] | 1.19 [1.00-1.41] |
|  | 38 to <63 | 16.43 to <28 | 1.20 [1.00-1.44] | 1.27 [1.07-1.51] |
|  | 38 to <63 | 28 to ≤48 | 1.34 [1.11-1.62] | 1.16 [0.96-1.39] |
|  | 63 to ≤120 | 1 to <7.75 | 1.04 [0.80-1.36] | 1.33 [1.05-1.68] |
|  | 63 to ≤120 | 7.75 to <16.43 | 1.29 [1.06-1.56] | 1.33 [1.10-1.59] |
|  | 63 to ≤120 | 16.43 to <28 | 1.41 [1.16-1.70] | 1.31 [1.09-1.57] |
|  | 63 to ≤120 | 28 to ≤48 | 1.24 [0.99-1.55] | 1.44 [1.16-1.78] |
| *Live Donor Recipients* |  |  |  |  |
|  | 10 to <23 | >0 to <0.42 | Ref. | Ref. |
|  | 10 to <23 | 0.42 to <1 | 1.34 [0.90-2.01] | 1.15 [0.75-1.76] |
|  | 10 to <23 | 1 to <3.04 | 1.49 [1.05-2.13] | 1.17 [0.81-1.69] |
|  | 10 to <23 | 3.04 to ≤24 | 0.95 [0.54-1.68] | 0.60 [0.30-1.17] |
|  | 23 to <35 | >0 to <0.42 | 1.30 [0.91-1.86] | 1.03 [0.71-1.49] |
|  | 23 to <35 | 0.42 to <1 | 1.32 [0.94-1.86] | 1.09 [0.77-1.54] |
|  | 23 to <35 | 1 to <3.04 | 1.31 [0.95-1.82] | 1.20 [0.86-1.68] |
|  | 23 to <35 | 3.04 to ≤24 | 1.17 [0.80-1.72] | 1.12 [0.76-1.65] |
|  | 35 to <60 | >0 to <0.42 | 1.35 [0.92-1.96] | 0.95 [0.64-1.42] |
|  | 35 to <60 | 0.42 to <1 | 1.34 [0.96-1.88] | 0.97 [0.68-1.37] |
|  | 35 to <60 | 1 to <3.04 | 1.47 [1.06-2.03] | 1.17 [0.84-1.64] |
|  | 35 to <60 | 3.04 to ≤24 | 1.13 [0.78-1.63] | 1.01 [0.69-1.46] |
|  | 60 to ≤120 | >0 to <0.42 | 1.52 [0.90-2.57] | 1.07 [0.63-1.81] |
|  | 60 to ≤120 | 0.42 to <1 | 1.67 [1.09-2.57] | 1.01 [0.63-1.63] |
|  | 60 to ≤120 | 1 to <3.04 | 1.55 [1.09-2.20] | 1.30 [0.91-1.86] |
|  | 60 to ≤120 | 3.04 to ≤24 | 1.64 [0.98-2.75] | 1.46 [0.91-2.35] |

Adjusted for donor characteristics: donor age, donor sex, donor race, donor body mass index category, donor hypertension, donor diabetes, and donor hepatitis C status; donor type: expanded donor criteria, donation after circulatory death (for deceased donors only); immunologic characteristics: number of human leukocyte antigen mismatches, panel reactive antibody category; surgical characteristics: cold ischemia time, warm ischemia time, transplant side; and recipient characteristics: age, sex, recipient race, body mass index category, dialysis vintage, cause of end stage renal disease, recipient diabetes, hypertension, coronary artery disease, cerebrovascular disease, peripheral vascular disease, prior malignancy, previous kidney transplant.

^a^ death with functioning graft

CI, confidence interval; OR, odds ratio

**Supplemental Table S5.** Association of WIT/CIT categories and delayed graft function

|  | **Warm Ischemia Time (minutes)** | **Cold Ischemia  Time (hours)** | **Delayed Graft Function  OR [95%CI]** |
| --- | --- | --- | --- |
| *Deceased Donor Recipients* |  |  |  |
|  | 10 to <25 | 1 to <7.75 | Ref. |
|  | 10 to <25 | 7.75 to <16.43 | 1.42 [1.17-1.71] |
|  | 10 to <25 | 16.43 to <28 | 2.05 [1.70-2.48] |
|  | 10 to <25 | 28 to ≤48 | 3.56 [2.81-4.50] |
|  | 25 to <38 | 1 to <7.75 | 1.04 [0.86-1.26] |
|  | 25 to <38 | 7.75 to <16.43 | 1.55 [1.30-1.84] |
|  | 25 to <38 | 16.43 to <28 | 2.19 [1.84-2.61] |
|  | 25 to <38 | 28 to ≤48 | 2.85 [2.37-3.44] |
|  | 38 to <63 | 1 to <7.75 | 1.19 [0.98-1.45] |
|  | 38 to <63 | 7.75 to <16.43 | 1.70 [1.43-2.02] |
|  | 38 to <63 | 16.43 to <28 | 2.27 [1.91-2.69] |
|  | 38 to <63 | 28 to ≤48 | 2.96 [2.47-3.54] |
|  | 63 to ≤120 | 1 to <7.75 | 1.26 [0.97-1.63] |
|  | 63 to ≤120 | 7.75 to <16.43 | 1.69 [1.40-2.04] |
|  | 63 to ≤120 | 16.43 to <28 | 2.53 [2.11-3.04] |
|  | 63 to ≤120 | 28 to ≤48 | 2.63 [2.12-3.24] |
| *Live Donor Recipients* |  |  |  |
|  | 10 to <23 | >0 to <0.42 | Ref. |
|  | 10 to <23 | 0.42 to <1 | 1.51 [0.77-2.94] |
|  | 10 to <23 | 1 to <3.04 | 1.86 [1.02-3.41] |
|  | 10 to <23 | 3.04 to ≤24 | 1.63 [0.70-3.75] |
|  | 23 to <35 | >0 to <0.42 | 1.49 [0.81-2.75] |
|  | 23 to <35 | 0.42 to <1 | 1.40 [0.78-2.51] |
|  | 23 to <35 | 1 to <3.04 | 1.70 [0.97-2.99] |
|  | 23 to <35 | 3.04 to ≤24 | 1.59 [0.85-2.99] |
|  | 35 to <60 | >0 to <0.42 | 1.66 [0.87-3.15] |
|  | 35 to <60 | 0.42 to <1 | 1.30 [0.72-2.35] |
|  | 35 to <60 | 1 to <3.04 | 1.94 [1.11-3.39] |
|  | 35 to <60 | 3.04 to ≤24 | 2.19 [1.21-3.96] |
|  | 60 to ≤120 | >0 to <0.42 | 2.49 [1.10-5.62] |
|  | 60 to ≤120 | 0.42 to <1 | 3.19 [1.62-6.29] |
|  | 60 to ≤120 | 1 to <3.04 | 2.67 [1.51-4.75] |
|  | 60 to ≤120 | 3.04 to ≤24 | 3.44 [1.80-6.57] |

CI, confidence interval; OR, odds ratio

**Supplemental Table S6.** Association between WIT/CIT categories and all-cause graft failure (clinical categories of WIT/CIT)

|  | **Warm Ischemia Time (minutes)** | **Cold Ischemia Time (hours)** | **Death or Graft Failure**  **HR [95%CI]**  ***Unadjusted Model*** | **Death or Graft Failure**  **HR [95%CI]**  ***Adjusted Model*** |
| --- | --- | --- | --- | --- |
| *Deceased Donor Recipients* |  |  |  |  |
|  | 10 to <30 | 1 to <12 | Ref. | Ref. |
|  | 10 to <30 | 12 to <24 | 1.02 [0.97-1.08] | 1.05 [0.98-1.12] |
|  | 10 to <30 | 24 to ≤48 | 1.12 [1.04-1.20] | 1.15 [1.06-1.26] |
|  | 30 to <60 | 1 to <12 | 1.04 [0.99-1.10] | 1.05 [0.99-1.12] |
|  | 30 to <60 | 12 to <24 | 1.13 [1.08-1.19] | 1.14 [1.08-1.21] |
|  | 30 to <60 | 24 to ≤48 | 1.21 [1.14-1.27] | 1.19 [1.12-1.27] |
|  | 60 to ≤120 | 1 to <12 | 1.19 [1.11-1.28] | 1.21 [1.12-1.31] |
|  | 60 to ≤120 | 12 to <24 | 1.26 [1.19-1.33] | 1.23 [1.15-1.31] |
|  | 60 to ≤120 | 24 to ≤48 | 1.31 [1.22-1.40] | 1.27 [1.17-1.37] |
| *Live Donor Recipients* |  |  |  |  |
|  | 10 to <30 | >0 to <1 | Ref. | Ref. |
|  | 10 to <30 | 1 to <2 | 1.25 [1.14-1.37] | 1.06 [0.94-1.19] |
|  | 10 to <30 | 2 to ≤24 | 1.16 [1.04-1.29] | 1.00 [0.87-1.15] |
|  | 30 to <60 | >0 to <1 | 1.04 [0.95-1.14] | 0.94 [0.85-1.05] |
|  | 30 to <60 | 1 to <2 | 1.32 [1.22-1.43] | 1.09 [0.99-1.20] |
|  | 30 to <60 | 2 to ≤24 | 1.26 [1.16-1.37] | 1.02 [0.92-1.13] |
|  | 60 to ≤120 | >0 to <1 | 1.22 [1.02-1.46] | 1.09 [0.90-1.33] |
|  | 60 to ≤120 | 1 to <2 | 1.37 [1.24-1.52] | 1.21 [1.04-1.41] |
|  | 60 to ≤120 | 2 to ≤24 | 1.48 [1.32-1.64] | 1.21 [1.01-1.46] |

Adjusted for donor characteristics: donor age, donor sex, donor race, donor body mass index category, donor hypertension, donor diabetes, and donor hepatitis C status; donor type: expanded donor criteria, donation after circulatory death (for deceased donors only); immunologic characteristics: number of human leukocyte antigen mismatches, panel reactive antibody category; surgical characteristics: cold ischemia time, warm ischemia time, transplant side; and recipient characteristics: age, sex, recipient race, body mass index category, dialysis vintage, cause of end stage renal disease, recipient diabetes, hypertension, coronary artery disease, cerebrovascular disease, peripheral vascular disease, prior malignancy, previous kidney transplant.

CI, confidence interval; HR, hazard ratio

**Supplemental Table S7.** Association between combined WIT/CIT and all-cause graft failure, including all values of WIT/CIT

|  | **Warm Ischemia Time**  **(minutes)** | **Cold Ischemia Time**  **(hours)** | **Relative Hazard, HR [95% CI]**  ***Adjusted Model*** |
| --- | --- | --- | --- |
| *Deceased Donor Recipients* |  |  |  |
|  | 0 to <17 | 1 to <7.75 | Ref. |
|  | 0 to <17 | 7.75 to <16.43 | 1.05 [0.93-1.19] |
|  | 0 to <17 | 16.43 to <28 | 1.17 [1.03-1.32] |
|  | 0 to <17 | 28 to ≤48 | 1.32 [1.11-1.56] |
|  | 17 to <36 | 1 to <7.75 | 0.97 [0.86-1.10] |
|  | 17 to <36 | 7.75 to <16.43 | 1.06 [0.94-1.18] |
|  | 17 to <36 | 16.43 to <28 | 1.12 [1.00-1.25] |
|  | 17 to <36 | 28 to ≤48 | 1.15 [1.01-1.31] |
|  | 36 to <63 | 1 to <7.75 | 0.98 [0.87-1.12] |
|  | 36 to <63 | 7.75 to <16.43 | 1.10 [0.98-1.23] |
|  | 36 to <63 | 16.43 to <28 | 1.15 [1.03-1.29] |
|  | 36 to <63 | 28 to ≤48 | 1.22 [1.08-1.37] |
|  | ≥63 | 1 to <7.75 | 1.12 [0.95-1.31] |
|  | ≥63 | 7.75 to <16.43 | 1.24 [1.10-1.40] |
|  | ≥63 | 16.43 to <28 | 1.29 [1.14-1.45] |
|  | ≥63 | 28 to ≤48 | 1.24 [1.08-1.43] |
| *Live Donor Recipients* |  |  |  |
|  | 0 to <4 | 0 to <0.5 | Ref. |
|  | 0 to <4 | 0.5 to <1 | 1.59 [1.10-2.30] |
|  | 0 to <4 | 1 to <3.48 | 1.29 [0.97-1.72] |
|  | 0 to <4 | ≥3.48 | 1.48 [1.01-2.18] |
|  | 4 to <33 | 0 to <0.5 | 1.16 [0.86-1.55] |
|  | 4 to <33 | 0.5 to <1 | 1.25 [0.94-1.67] |
|  | 4 to <33 | 1 to <3.48 | 1.30 [0.98-1.72] |
|  | 4 to <33 | ≥3.48 | 1.06 [0.78-1.44] |
|  | 33 to <60 | 0 to <0.5 | 1.11 [0.82-1.50] |
|  | 33 to <60 | 0.5 to <1 | 1.17 [0.88-1.56] |
|  | 33 to <60 | 1 to <3.48 | 1.35 [1.02-1.79] |
|  | 33 to <60 | ≥3.48 | 1.09 [0.80-1.47] |
|  | ≥60 | 0 to <0.5 | 1.26 [0.86-1.84] |
|  | ≥60 | 0.5 to <1 | 1.41 [0.98-2.01] |
|  | ≥60 | 1 to <3.48 | 1.48 [1.10-1.98] |
|  | ≥60 | ≥3.48 | 1.66 [1.13-2.43] |

Adjusted for donor characteristics: donor age, donor sex, donor race, donor body mass index category, donor hypertension, donor diabetes, and donor hepatitis C status; donor type: expanded donor criteria, donation after circulatory death (for deceased donors only); immunologic characteristics: number of human leukocyte antigen mismatches, panel reactive antibody category; surgical characteristics: cold ischemia time, warm ischemia time, transplant side; and recipient characteristics: age, sex, recipient race, body mass index category, dialysis vintage, cause of end stage renal disease, recipient diabetes, hypertension, coronary artery disease, cerebrovascular disease, peripheral vascular disease, prior malignancy, previous kidney transplant.

CI, confidence interval; HR, Hazard Ratio

**Supplemental Table S8.** Association of combined WIT/CIT with all-cause graft failure after exclusion of donation after circulatory death recipients

|  | **Warm Ischemia Time (minutes)** | **Cold Ischemia Time (hours)** | **Relative Hazard  HR [95% CI]** |
| --- | --- | --- | --- |
| *Data-driven approach* |  |  |  |
|  | 10 to <25 | 1 to <7.75 | Ref. |
|  | 10 to <25 | 7.75 to <16.43 | 1.08 [0.94-1.24] |
|  | 10 to <25 | 16.43 to <28 | 1.11 [0.97-1.27] |
|  | 10 to <25 | 28 to ≤48 | 1.23 [1.01-1.49] |
|  | 25 to <38 | 1 to <7.75 | 1.02 [0.89-1.17] |
|  | 25 to <38 | 7.75 to <16.43 | 1.12 [0.99-1.28] |
|  | 25 to <38 | 16.43 to <28 | 1.20 [1.06-1.36] |
|  | 25 to <38 | 28 to ≤48 | 1.22 [1.06-1.40] |
|  | 38 to <63 | 1 to <7.75 | 1.06 [0.93-1.22] |
|  | 38 to <63 | 7.75 to <16.43 | 1.17 [1.03-1.33] |
|  | 38 to <63 | 16.43 to <28 | 1.23 [1.09-1.40] |
|  | 38 to <63 | 28 to ≤48 | 1.23 [1.10-1.43] |
|  | 63 to ≤120 | 1 to <7.75 | 1.20 [1.01-1.43] |
|  | 63 to ≤120 | 7.75 to <16.43 | 1.31 [1.15-1.50] |
|  | 63 to ≤120 | 16.43 to <28 | 1.35 [1.18-1.54] |
|  | 63 to ≤120 | 28 to ≤48 | 1.35 [1.16-1.58] |
| *Clinical approach* |  |  |  |
|  | 10 to <30 | 1 to <12 | Ref. |
|  | 10 to <30 | 12-<24 | 1.05 [0.98-1.12] |
|  | 10 to <30 | 24-<48 | 1.15 [1.05-1.25] |
|  | 30 to <60 | 1 to <12 | 1.05 [0.99-1.12] |
|  | 30 to <60 | 12-<24 | 1.14 [1.08-1.20] |
|  | 30 to <60 | 24-<48 | 1.19 [1.12-1.27] |
|  | 60-<120 | 1 to <12 | 1.21 [1.12-1.32] |
|  | 60-<120 | 12-<24 | 1.23 [1.15-1.31] |
|  | 60-<120 | 24-<48 | 1.27 [1.69-1.37] |

Adjusted for donor characteristics: donor age, donor sex, donor race, donor body mass index category, donor hypertension, donor diabetes, and donor hepatitis C status; donor type: expanded donor criteria (for deceased donors only); immunologic characteristics: number of human leukocyte antigen mismatches, panel reactive antibody category; surgical characteristics: cold ischemia time, warm ischemia time, transplant side; and recipient characteristics: age, sex, recipient race, body mass index category, dialysis vintage, cause of end stage renal disease, recipient diabetes, hypertension, coronary artery disease, cerebrovascular disease, peripheral vascular disease, prior malignancy, previous kidney transplant.

CI, confidence interval; HR, Hazard Ratio

**Supplemental Table S9.** Association of combined WIT/CIT and all-cause graft failure in the imputed data set (multiple imputation using chained equations)

a) Data-driven approach

|  | **Warm Ischemia Time (minutes)** | **Cold Ischemia Time (hours)** | **Hazard Ratio  [95% CI]** |
| --- | --- | --- | --- |
| *Deceased Donor Recipients* |  |  |  |
|  | 10 to <25 | 1 to <7.75 | Ref. |
|  | 10 to <25 | 7.75 to <16.43 | 1.00 [0.89-1.14] |
|  | 10 to <25 | 16.43 to <28 | 1.16 [1.02-1.31] |
|  | 10 to <25 | 28 to ≤48 | 1.25 [1.06-1.47] |
|  | 25 to <38 | 1 to <7.75 | 1.09 [0.96-1.24] |
|  | 25 to <38 | 7.75 to <16.43 | 1.08 [0.97-1.21] |
|  | 25 to <38 | 16.43 to <28 | 1.24 [1.11-1.39] |
|  | 25 to <38 | 28 to ≤48 | 1.19 [1.05-1.34] |
|  | 38 to <63 | 1 to <7.75 | 1.10 [0.97-1.25] |
|  | 38 to <63 | 7.75 to <16.43 | 1.03 [0.92-1.16] |
|  | 38 to <63 | 16.43 to <28 | 1.20 [1.07-1.34] |
|  | 38 to <63 | 28 to ≤48 | 1.38 [1.23-1.56] |
|  | 63 to ≤120 | 1 to <7.75 | 1.03 [0.87-1.22] |
|  | 63 to ≤120 | 7.75 to <16.43 | 1.36 [1.21-1.54] |
|  | 63 to ≤120 | 16.43 to <28 | 1.33 [1.18-1.50] |
|  | 63 to ≤120 | 28 to ≤48 | 1.36 [1.18-1.56] |
| *Live Donor Recipients* |  |  |  |
|  | 10 to <23 | >0 to <0.42 | Ref. |
|  | 10 to <23 | 0.42 to <1 | 1.33 [1.19-1.50] |
|  | 10 to <23 | 1 to <3.04 | 1.63 [1.48-1.80] |
|  | 10 to <23 | 3.04 to ≤24 | 1.33 [1.15-1.54] |
|  | 23 to <35 | >0 to <0.42 | 1.31 [1.18-1.45] |
|  | 23 to <35 | 0.42 to <1 | 1.34 [1.22-1.49] |
|  | 23 to <35 | 1 to <3.04 | 1.51 [1.38-1.66] |
|  | 23 to <35 | 3.04 to ≤24 | 1.22 [1.10-1.36] |
|  | 35 to <60 | >0 to <0.42 | 1.41 [1.27-1.57] |
|  | 35 to <60 | 0.42 to <1 | 1.35 [1.23-1.49] |
|  | 35 to <60 | 1 to <3.04 | 1.67 [1.52-1.83] |
|  | 35 to <60 | 3.04 to ≤24 | 1.49 [1.35-1.65] |
|  | 60 to ≤120 | >0 to <0.42 | 1.56 [1.33-1.82] |
|  | 60 to ≤120 | 0.42 to <1 | 1.73 [1.53-1.96] |
|  | 60 to ≤120 | 1 to <3.04 | 1.68 [1.53-1.85] |
|  | 60 to ≤120 | 3.04 to ≤24 | 1.59 [1.41-1.79] |

*b) Clinical approach*

|  | **Warm Ischemia Time (minutes)** | **Cold Ischemia Time (hours)** | **Hazard Ratio  [95% CI]** |
| --- | --- | --- | --- |
| *Deceased Donor Recipients* |  |  |  |
|  | 10 to <30 | 1 to <12 | Ref. |
|  | 10 to <30 | 12-<24 | 1.16 [1.09-1.24] |
|  | 10 to <30 | 24-<48 | 1.21 [1.12-1.31] |
|  | 30 to <60 | 1 to <12 | 1.14 [1.07-1.21] |
|  | 30 to <60 | 12-<24 | 1.18 [1.11-1.25] |
|  | 30 to <60 | 24-<48 | 1.33 [1.25-1.41] |
|  | 60-<120 | 1 to <12 | 1.11 [1.02-1.21] |
|  | 60-<120 | 12-<24 | 1.39 [1.30-1.48] |
|  | 60-<120 | 24-<48 | 1.43 [1.33-1.55] |
| *Live Donor Recipients* |  |  |  |
|  | 10 to <30 | >0 to <1 | Ref. |
|  | 10 to <30 | 1 to <2 | 1.17 [1.12-1.22] |
|  | 10 to <30 | 2 to <24 | 1.20 [1.14-1.26] |
|  | 30 to <60 | >0 to <1 | 1.06 [1.01-1.10] |
|  | 30 to <60 | 1 to <2 | 1.24 [1.19-1.28] |
|  | 30 to <60 | 2 to <24 | 1.27 [1.22-1.32] |
|  | >=60 | >0 to <1 | 1.31 [1.21-1.41] |
|  | >=60 | 1 to <2 | 1.33 [1.27-1.40] |
|  | >=60 | 2 to <24 | 1.26 [1.20-1.33] |

Adjusted for donor characteristics: donor age, donor sex, donor race, donor body mass index category, donor hypertension, donor diabetes, and donor hepatitis C status; donor type: expanded donor criteria, donation after circulatory death (for deceased donors only); immunologic characteristics: number of human leukocyte antigen mismatches, panel reactive antibody category; surgical characteristics: cold ischemia time, warm ischemia time, transplant side; and recipient characteristics: age, sex, recipient race, body mass index category, dialysis vintage, cause of end stage renal disease, recipient diabetes, hypertension, coronary artery disease, cerebrovascular disease, peripheral vascular disease, prior malignancy, previous kidney transplant.

CI, confidence interval; HR, Hazard Ratio


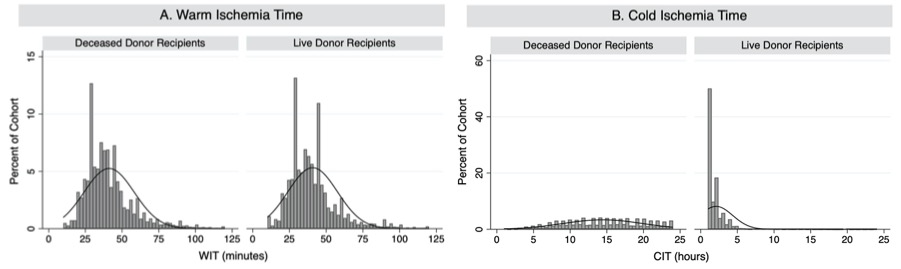


**Supplemental Figure S1.** a) Distribution of warm ischemia time and b) cold ischemia time for live donor and deceased donor recipients. Warm ischemia times of <10 minutes and >120 minutes and cold ischemia times of <1 hour and >24 hours for deceased donor recipients and 0 hours for live donor recipients were excluded.

CIT, cold ischemia time; WIT, warm ischemia time
